# Editorial for the Special Issue on Optics and Photonics in Micromachines

**DOI:** 10.3390/mi14061102

**Published:** 2023-05-23

**Authors:** Cuifang Kuang, Wei Zhao

**Affiliations:** 1 College of Optical Science and Engineering, Zhejiang University, Hangzhou 310027, China; cfkuang@zju.edu.cn; 2 State Key Laboratory of Photon-Technology in Western China Energy, International Scientific and Technological Cooperation Base of Photoelectric Technology and Functional Materials and Application, Institute of Photonics and Photon Technology, Northwest University, Xi’an 710127, China

Micromachines, as a platform for manipulation, assembling, detection and imaging, is a typical interdisciplinary field related to broad areas, e.g., electric and electronics, mechanics, optics and photonics, chemistry and biomedicine, etc. As a major approach in micromachines, on the one hand, optics and photonics are frequently applied in the design and functionalization of novel microdevices, for instance, high-performance metalenses [1], integratable laser sources [2], Microelectromechanical systems (MEMS) mirrors [3], etc. On the other hand, they are also ubiquitous in the applications of microdevices and research in microscale phenomena, e.g., sensing [4,5], imaging [6,7,8], characterizing [9,10], etc.

In this Special Issue, we are glad to collect 15 research articles covering a broad area, including optical field modulation [11], laser fabrication techniques [12,13], optical measurement [14,15,16,17,18], on-chip photonic devices [19,20,21,22], super-resolution imaging [23,24], and related theoretical [25] investigations.

Among the research articles, three of them are related to the fabrication techniques of microdevices. Liu et al. [11] advanced a noniterative method—Circular-Sectorial Phase Segmentation—for designing the holographic phase maps of spatial light modulators to realize multi-focus arrays. The experimental results indicate that the method can be applied to the parallel fabrication of nanostructures with flexibility and uniformity. The research provides an effective approach for light field modulation, which could be applied to laser fabrication, optofluidics and biophotonics. Wen et al. [12] showed that by spatial light modulation, a fascinating and tightly focused multi-ramp helico-conical optical beam can be generated to directly fabricate chiral 3D structures on a microscale. Xu et al. [13] theoretically evaluated the influence of the inevitable fabrication error on dielectric metalenses consisting of GaAs nano-bricks on CaF_2_ substrate. One important observation that may attract the interest of the industry field is that the full width at half maximum (FWHM) of the beam, which is focused by the metalens, is insensitive to fabrication errors.

Four of them correspond to the development of on-chip and integrable photonic devices, which is a major branch of micromachines. Armghan et al. [19] employed a machine-learning method to assist in the design of VCSEL for high-yield optical wireless networks. Chen et al. [20] developed a plenoptic camera based on an electrically tunable liquid crystal with a hexagonal-period liquid crystal array for all-in-focus polarimetric imaging, dedicated to providing more optical information for both scientific and daily applications. Yue et al. [22] proposed a method to integrate 2D optical phased arrays with an on-chip multi-quantum-well laser array heterogeneously based on a silicon photonic platform. By employing four key components, including a multi-quantum-well laser array, electro-optic switch array, phase-shifter array and a Bragg-waveguide-grating antenna array, the device can realize 2D spatial beam scanning with a large steering range of 88.4° × 18°. Note, the device is only 8 mm × 3 mm, with a modulation rate as low as 2.5 ps. In optics, sometimes a fast optical focusing is required for the temporal modulation of a light beam. For this purpose, Ilkhechi et al. [21] proposed a deformable MEMS mirror for ultrafast optical focusing. The device can reach a 4.9 MHz modulation rate for 1 mm focus movement, with response times smaller than 5 μs. Although these specifications are still 1–2 orders weaker than that of an acousto-optic modulator, the device will not cause the degradation of beam quality and significant loss of beam power. Therefore, it could attract more applications in many scenes, e.g., super-resolution imaging.

Optical diagnostic techniques are another major branch in micromachines and have broad applications. Liao et al. [16] developed optical coherence tomography (OCT), named full-range OCT, by applying a low-cost voice coil motor. A two-fold increment of accessible depth range was achieved, relative to the conventional OCT. Medina et al. [14] developed a novel optical method by combining fluorescence and near-infrared imaging to complementarily measure the film thickness and concentration of a falling film, which has a speed of up to 500 mm/s. Zhao et al. [17] built an AC electrokinetic microchip and an optical system for the point-of-care testing (POCT) of bacteria lysis. Zhang et al. [15] adopted confocal Raman spectroscopy to reveal the underlying mechanism of a dynamic interaction between doxorubicin (DOX) and DNA on a single-molecule level. They showed that DOX can interact with all four bases of DNA molecules and change the conformation of DNA according to two routes: “DOX-DNA acts to form a complex, and DOX-DOX acts to form a multimer.” The former tends to compress DNA molecules, while the latter tends to decompress DNA molecules. Since DOX and its derivatives are commonly used in anticancer therapies, understanding the interaction between DOX and DNA is fundamentally important for revealing the minor effects of DOX. Furthermore, Tian et al. [18] proposed an effective way to improve the reconstructive quality of a digital hologram by a two-step converging spherical wave.

The imaging process method is also important in the development of optical diagnostic techniques. There are another two research articles related directly to super-resolution imaging. One used the traditional method [23] by combining Lucy–Richardson deconvolution and discrete wavelet methods, and the other one used an A-net deep learning network [24]. Both show apparent improvement in the spatial resolution and SNR of biological images. By the post-processing methods, the raw confocal microscopy images with spatial resolution over the diffraction limit can be enhanced to below the diffraction limit. There is also another machine-learning-based study on the optimization of CW laser [25].

We hope all the collected investigations in optics and photonics can be of interest to both the scientific community and industry in the field of micromachines. Furthermore, I would like to take this opportunity to appreciate all of the authors’ contributions. I also appreciate all the reviewers for dedicating their time to help improve the submitted papers and elevate the quality of the Special Issue.
